# Aging is associated with increased regulatory T-cell function

**DOI:** 10.1111/acel.12191

**Published:** 2014-02-25

**Authors:** Sanjay K Garg, Colin Delaney, Tomomi Toubai, Amiya Ghosh, Pavan Reddy, Ruma Banerjee, Raymond Yung

**Affiliations:** 1 Division of Geriatrics and Palliative MedicineAnn Arbor, MI-48109, USA; 2 Division of Hematology and Oncology, Department of Internal MedicineAnn Arbor, MI-48109, USA; 3 Department of Biological Chemistry, University of Michigan Medical SchoolAnn Arbor, MI-48109, USA; 4 Geriatrics Research, Education and Clinical Care Center (GRECC), VA Ann Arbor Healthcare System, 2215 Fuller RoadAnn Arbor, MI-48105, USA

**Keywords:** aging, epigenetics, methylation, redox, regulatory T cell

## Abstract

Regulatory T-cell (Treg, CD4^+^CD25^+^) dysfunction is suspected to play a key role in immune senescence and contributes to increased susceptibility to diseases with age by suppressing T-cell responses. FoxP3 is a master regulator of Treg function, and its expression is under control of several epigenetically labile promoters and enhancers. Demethylation of CpG sites within these regions is associated with increased FoxP3 expression and development of a suppressive phenotype. We examined differences in FoxP3 expression between young (3–4 months) and aged (18–20 months) C57BL/6 mice. DNA from CD4^+^ T cells is hypomethylated in aged mice, which also exhibit increased Treg numbers and FoxP3 expression. Additionally, Treg from aged mice also have greater ability to suppress effector T-cell (Teff) proliferation *in vitro* than Tregs from young mice. Tregs from aged mice exhibit greater redox remodeling–mediated suppression of Teff proliferation during coculture with DCs by decreasing extracellular cysteine availability to a greater extent than Tregs from young mice, creating an adverse environment for Teff proliferation. Tregs from aged mice produce higher IL-10 levels and suppress CD86 expression on DCs more strongly than Tregs from young mice, suggesting decreased T-cell activity. Taken together, these results reveal a potential mechanism of higher Treg-mediated activity that may contribute to increased immune suppression with age.

## Introduction

It is generally accepted that aging is associated with altered immune response including decay in Th1-Th2-mediated immunity, diminished naïve T-cell-mediated de novo responses and an altered memory T-cell compartment (Raynor *et al*., 2012). Consequently, immune senescence is associated with higher susceptibility to infections, autoimmunity, and cancer in the elderly (Richardson, 2002; Mendez *et al*., 2004; Sharma *et al*., 2006). Moreover, while aging has been reported to be associated with increased regulatory T-cell (Treg, CD4^+^ CD25^+^) numbers, there is little information about its functional consequences on immune suppression.

Tregs can be defined by the expression of the forkhead/winged-helix family of transcription factor (FoxP3) that is both necessary and sufficient for Treg development (Hori *et al*., 2003). Two major populations of Tregs exist: the so-called natural (nTreg) and inducible (iTreg) Tregs. nTregs are produced in the thymus, while iTregs differentiate extrathymically in secondary lymphoid organs in response to antigen exposure (Huehn *et al*., 2009). Tregs maintain peripheral tolerance and keep autoreactive cells at bay. However, excess Treg activity may lead to increased susceptibility to infection, neurodegenerative diseases, and cancer (Richardson, 2002; Mendez *et al*., 2004; Sharma *et al*., 2006).

Although recent studies have shown that aging is associated with increased Treg numbers, the underlying mechanism of this phenomenon is unknown. One potential mechanism might be age-related alteration in epigenetic programming affecting Tregs. Epigenetic regulation of gene expression occurs via chemical modifications, such as histone acetylation and methylation, without alterations in the nucleotide sequence in the genome (Delaney *et al*., 2012). DNA methylation is associated with gene silencing, while demethylation promotes gene expression. T-cell DNA is hypomethylated in older relative to young individuals (Agrawal *et al*., 2010). The FoxP3 locus possesses several epigenetically labile regulatory regions which are differentially methylated in subsets of CD4^+^ cells (Huehn *et al*., 2009). For example, it is known that naïve CD4^+^CD25^−^ T cells, activated CD4^+^ cells, and TGF-β-induced Treg cells are methylated at CpG dinucleotides upstream of the FoxP3 enhancer but demethylated in nTregs (Lal & Bromberg, 2009). However, it is not known whether a differential methylation pattern exists at the FoxP3 locus between young and old mice as a potential mechanism underlying higher Treg expression.

Induction of optimal effector T-cell (Teff) responses depends on their interaction with costimulatory molecules (e.g., CD80/86) on dendritic cells (DCs). Tregs suppress DC activity by downregulating these molecules (Cederbom *et al*., 2000). Tregs diminish the ability of antigen-presenting cells (APCs) including DCs to form stable contacts with responding T cells, thereby interfering with T-cell activation. Recently, it was shown that DCs from older mice express lower CD86 *in vivo* relative to DCs from younger mice (Chiu *et al*., 2007). However, it is unknown whether this lower CD86 expression is due to age-associated decline or if Tregs from aged animal have greater capacity to downregulate CD86 expression or both.

In addition to the classical T-cell activation pathway involving TCR-antigen-MHC II interaction, costimulatory and cytokines signals, it was recently shown that T-cell activation and proliferation is dependent on a DC-mediated provision of a reducing extracellular microenvironment (Yan *et al*., 2009). DC/T-cell interactions induce cystine uptake in DCs via the xc- cystine transporter. DCs uptake cystine for intracellular glutathione (GSH) biosynthesis, and they provide extracellular cysteine that shifts the redox potential in the reducing direction, which is favorable for T-cell proliferation (Yan *et al*., 2009; Yan & Banerjee, 2010). As naïve T cells are unable to efficiently transport cysteine, the extracellular cysteine made available by DCs is essential for supporting GSH synthesis (Garg *et al*., 2011) which is essential for cell cycle progression (Messina & Lawrence, 1989; Suthanthiran *et al*., 1990), cytotoxic T-cell activity (Grimble, 2006), and T-cell signal transduction (Staal, 1998). Hence, by modulating extracellular cysteine levels, DCs support intracellular GSH synthesis in T cells and adjust the redox potential to favor T-cell activation and proliferation (Yan & Banerjee, 2010). Large increases in nonprotein thiols in lymphoid tissues have been observed following immunization (Castellani *et al*., 2008), demonstrating the physiological relevance of the redox microenvironment in immune function. Tregs interfere with this process by suppressing extracellular cysteine accumulation leading in turn to the suppression of T-cell activation and proliferation (Yan *et al*., 2009). The differential ability of Tregs from young vs. old mice to suppress the immune response by influencing the extracellular redox potential is not known.

In this study, we confirm that aged mice have higher levels of Tregs compared with young mice. The higher Treg numbers are associated with hypomethylation of the upstream FoxP3 enhancer, resulting in higher FoxP3 mRNA and protein level. We also demonstrate that Tregs from old mice produce more IL-10, downregulate the expression of the costimulatory CD86 on DCs, perturb the redox-mediated microenvironment and cause greater suppression of T-cell proliferation in comparison with Treg from young mice. To our knowledge, this is the first study reporting differences in Treg activity between young and old mice and offers a potential mechanism by which Treg activity contributes to immune senescence in old age.

## Experimental procedures

### Mice

Male young adult mice (3–4 months) were purchased from Harlan Laboratories (Indianapolis, IN, USA), while old (18–20 months) C57BL/6 mice were purchased from the National Institute on Aging (NIA). After arrival, animals were put on an *ad libitum* diet for at least 1 week prior to experiments. All mice were maintained in a pathogen-free environment provided by the Unit for Laboratory Medicine (ULAM) at the University of Michigan. Procedures involving the animals and their care were approved by the Committee on the Use and Care of Animals (UCUCA) at the University of Michigan Medical School.

### Cell preparation

(i) *DC isolation and differentiation*-bone marrow cells from C57BL/6 mice were depleted of red blood cells and plated at 2 × 10^6^ cells mL^−1^ for 7 days in DC medium (DMEM supplemented with 100 μg mL^−1^ penicillin and streptomycin, 2 mm L-glutamine, 50 μm 2-mercaptoethanol (2-ME), 1 mm pyruvate, 1:100 nonessential amino acids, and 10% heat-inactivated fetal bovine serum) containing recombinant murine rGM-CSF (20 ng mL^−1^, R&D Systems, Minnneapolis, MN, USA) and rIL-4 (20 ng mL^−1^, R&D Systems) (Yan *et al*., 2009). Culture medium was changed, and floating cells were discarded every 2 days. To check the purity of DCs, cells were labeled with anti-CD11c (1:1000 dilution) monoclonal antibodies (BD). The population of antibody-positive cells under FACS was estimated to be ≥ 85% (data not shown). Immature bone marrow–derived DCs were harvested at day 7 and used as APCs in the coculture experiments with T cells; (ii) *T-cell purification-*CD3^+^, CD4^+^, CD4^+^ CD25^−^ (Teff) and CD4^+^ CD25^+^ (Treg) T cells were isolated using the MACS microbeads technology (Miltenyi Biotec, Auburn, CA, USA) from the spleen and lymph node (LN) using negative and/or positive selection, according to the manufacturer’s instructions. Purity of the isolated cells was assessed by flow cytometric analysis by staining with FITC-conjugated anti-CD3 or PE-Cy5 anti-CD4 or isotype rat IgG2bAbs or rat IgG2a, respectively (BD). CD3^+^, CD4^+^, and Teff purity was consistently above 90%. Treg purity was between 80–90%. Isolated T cells were used either for *in vitro* coculture experiments, surface staining, protein or nucleic acid isolation as described below.

### Culture conditions

Dendritic cells (1 × 105/well) were cocultured in 48-well plates with or without Teff cells at 1:4 ratio in the presence or absence of Treg cells at 1:4:2 and 1:4:4 (DC/Teff/Treg) ratio for up to 72 h at 37 °C in a 5% CO2 incubator in T-cell medium (RPMI medium supplemented with 100 μg mL^−1^ penicillin and streptomycin, 2 mm L-glutamine, 50 μm 2-mercaptoethanol and 2.5% heat-inactivated fetal bovine serum) in the presence of plate-bound anti-CD3 antibody (1 μg mL^−1^, BD). At indicated time points (24, 72 h), aliquots of conditioned media were collected for extracellular thiol measurement by HPLC. Cells were harvested at 72 h for intracellular GSH measurement and Western blot analysis.

### Metabolite analysis

To measure extracellular thiols (cysteine and cystine), aliquots of culture supernatants were mixed with an equal volume of metaphosphoric acid solution (16.8 mg mL^−1^ HPO3, 2 mg mL^−1^ EDTA and 9 mg mL^−1^ NaCl) and vortexed. Proteins were sedimented by centrifugation at ~14 000 × ***g*** for 10 min at 4 °C. Metabolites in protein-free extracts were derivatized with monoiodoacetic acid (7 mm) followed by mixing with an equal volume of 2,4-dinitrofluorobenzene solution (1.5% v/v in absolute ethanol). Samples were separated by HPLC using u-Bondapak-NH2 300 × 3.9 mm column (Waters) with a methanol/acetate gradient and the cysteine and cystine peak was collected as described previously (Garg *et al*., 2008). The concentration of individual thiols was determined by comparing the integrated peak areas with standard curves for each compound. The contribution of metabolites in media blank was subtracted from the final values. To measure the concentration of intracellular GSH, cultured cells were washed with ice-cold PBS and harvested in PBS (50 μL). An aliquot of the cell suspension in PBS was mixed with an equal volume of metaphosphoric acid, vortexed, followed by monoiodoacetic acid derivatization, 2,4-dinitrofluorobenzene reaction and HPLC as described previously. Intracellular GSH concentrations were normalized to total protein concentration in each sample (Garg *et al*., 2006).

### Global methylation analysis

Flow cytometric measurement of global methylation was performed as described previously (Delaney *et al*., 2012). Briefly, 2.5 × 105 freshly harvested T cells were stained with FITC anti-CD3 Abs and PE-Cy5 anti-CD4, or isotypes, then fixed in Cytofix/Cytoperm (BD) and permeabilized using PBS supplemented with 0.1% saponin, 1% FBS, and 0.1% sodium azide. Cells were treated with RNase A to eliminate the potential for the detection of 5-methylcytidine in tRNA. Cells were treated with anti-5-methylcytidine (Acris Antibodies, San Diego, CA, USA), washed, then incubated with Anti-mouse IgG1-PE or isotype rat-IgG1κ (BD). Flow cytometry was performed immediately or within 24 h of permeabilization using a FACScaliber machine (BD). Results were analyzed with FCS Express software (de novo Software, Los Angeles, CA, USA).

### Regulatory T-cell (Treg) staining

Freshly isolated splenocytes were stained with PE-Cy5 anti-CD4 and PE-anti-CD25 or isotypes rat IgG2a,κ and rat IgM,κ, respectively, for 10 min at 4 °C (BD). Cells were then fixed and permeabilized using the Mouse FoxP3 Buffer Set and then incubated with Alexa Fluor 488 anti-FoxP3 or isotype rat IgG2baccording to manufacturer’s instructions (BD). Flow cytometry was performed immediately or within 24 h of permeabilization using a FACScaliber machine. 1 × 105 gated events were recorded. Results were analyzed with FCS Express software.

### Nucleic acid isolation

Total RNA was isolated from freshly isolated splenic T cells using RNeasy technology (Qiagen, Valencia, CA, USA) according to the manufacturer’s instructions. Carryover DNA contaminants were removed using the RNase-Free DNase Set. Genomic DNA was isolated using GenElute Mammalian Genomic DNA Miniprep Kit (Sigma, St. Louis, MO, USA). Isolated DNA and RNA were quantified using the NanoDrop spectrophotometer.

### Bisulfite treatment of T-cell genomic DNA and pyrosequencing of FoxP3 promoter and enhancer regions-

Sodium bisulfite treatment of 1 μg genomic DNA was performed with the EZ DNA Methylation Gold Kit (Zymo Research, Irvine, CA, USA) following the manufacturer’s instructions. After purification, the bisulfite-treated DNA was stored at −20 °C. Pyrosequencing assays were designed using PSQ Assay Design Software (Biotage, Charlotte, NC, USA). PCR conditions were 95 °C for 15 min, then 55 cycles of 95 °C for 15 s, 60 °C for 15 s, and 72 °C for 30 s using the Pyromark PCR kit (Qiagen). Unmodified and biotinylated primers were used at a final concentration of 0.2 μm and 0.1 μm, respectively. The biotinylated strands of the amplicon were purified using streptavidin–Sepharose beads (GE). We used reaction PyroGold reagents and manufacturer’s recommended conditions on the Pyromark MD platform using Pyro CpG software (Biotage). Primer sequences: FoxP3 proximal promoter Forward 5′-TTTTGTGGTGAGGGGAAGAA–3′ Reverse 5′–BIOTIN-ACCCTCAATACCTCTCTACCACTT–3′ Sequencing primer 5′– AAAATTGGATTATTAGAAG– 3′; FoxP3 upstream enhancer Forward 5′–BIOTIN-AATGTGGGTATTAGGTAAAATTTTT–3′ Reverse and sequencing primer B 5′– AACCCTAAAACTACCTCTAAC–3′ Sequencing primer A 5′–AAAACTTTTCCCAAACCCTCTC –3′.

### Western Blot analysis

T cells were separated from adherent DCs from the coculture wells by gentle pipetting and washing with PBS. Cells were washed two times with cold PBS and lysed by suspending in lysis buffer as described previously (Garg *et al*., 2006). FoxP3 (Abcam, Cambridge, MA, USA), CD86 and CD80 (R&D systems) and actin (Santa Cruz Biotechnology, Dallas, TX, USA) levels in cell extracts were detected using the respective primary and secondary antibodies and detected using the chemiluminescent horseradish peroxidase system (Pierce, Rockford, IL, USA) as described previously (Garg *et al*., 2006). The intensity of protein bands was quantified using the Image J software and normalized to the actin level in the same sample.

### Alpha-ELISA

Tregs from young and old mice were cocultured with DC and Teff in 1:4:2 and 1:4:4 (DC/Teff/Treg) ratios in the presence of anti-CD3 antibody for 72 h. In a separate experiment, Treg from young and old mice were cocultured with Teff from young and old mice at 1:1 ratio in the presence of plate-bound anti-CD3 (1 μg mL^−1^) and soluble anti-CD28 antibody (1 μg mL^−1^, BD). After 72 h, conditioned media were collected, and IL-10 concentration was measured by using alpha-ELISA (Perkin Elmer, Waltham, MA, USA) as per manufacturer’s instructions.

### *In vitro* suppression assay

CD4^+^ CD25^+^ Treg cells and CD4^+^ CD25^−^ Teff cells were isolated first using EasySep Mouse CD4^+^ T-Cell Isolation Kit (Stem Cell), then enriched cells were labeled with PE-Cy5-anti-CD4 and PE-anti-CD25 (BD) and flow sorted on a FACS Vantage (BD). Dendritic cells (DCs) were generated from bone marrow (BM) from either syngeneic (C57BL/6) or allogeneic (BALB/C) mouse strain as previously described (Yan *et al*., 2009). CD11c^+^ DCs from both conditions were purified with mouse CD11c MicroBeads Kit as per manufacturer instructions (Miltenyi biotec). Of 4 × 10^4^ Teff cells from 3–4 months old wild-type C57BL/6 were incubated in the indicated ratios in the T-cell media with Tregs from young and old mice in the presence of 5 × 103 CD11c^+^ DCs for ~5 days in round bottom 96-well plates. Media were supplemented with 1 μg mL^−1^ anti-CD3e (BD) when coculturing syngeneic BM-derived and splenic DCs. Anti-IL10 neutralization antibodies or N-acetyl cysteine (NAC), when used, was administered as a bolus to a final concentration of 10 μg mL^−1^ or 2 mm, respectively, at the start of coculture. Incorporation of 3H-thymidine (1 μCi/well, Perkin Elmer) by proliferating cells was measured during the last 18 h of culture.

### Statistical analysis

Mean and SEM were calculated using Microsoft Excel application software. Statistical significance was determined using one-tailed Student’s *t*-test for single comparison. *P* values of < 0.05 were considered significant.

## Results

### T cells from old mice are hypomethylated relative to T cells from young mice

**Figure 1:**
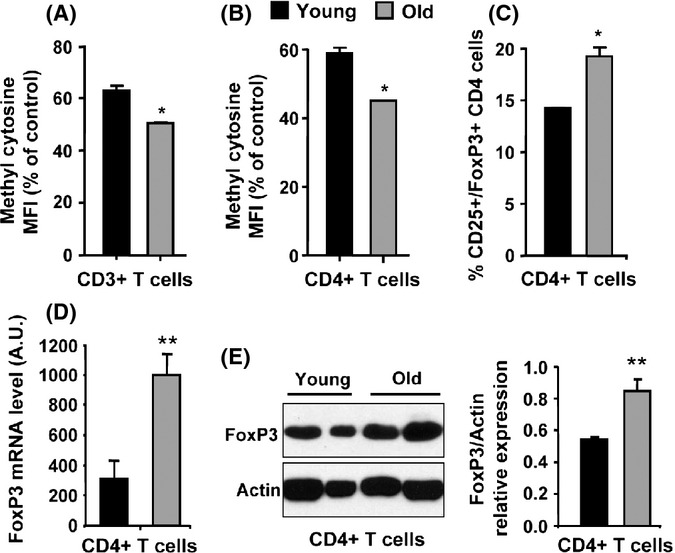
Global T-cell methylation decreases and FoxP3 expression increases during normal aging. Resting CD3^+^ T cells (A) or CD4^+^ cells (B) isolated from young (6–8 week) and old (18–20 week) mice were stained with antimethylcytidine antibody and subjected to FACS analysis. Resting CD4^+^ T cells from young and old mice were harvested. (C) Cells were stained with anti-CD4, anti-CD25, anti-FoxP3 antibody and subjected to FACS analysis. (D) Total RNA was isolated and FoxP3 mRNA was measured via real-time qPCR. (E) Cytoplasmic proteins were used to measure the level of FoxP3 protein via Western blot analysis and normalized to the level of actin in that sample. Results are mean ± SEM of four experiments, each experiment using cells pooled from at least three animals per age group; *P < 0.05, **P < 0.01.

T cell’s DNA from aged individuals are hypo-methylated relative to T cells from young individuals (Agrawal *et al*., 2010). To validate previous studies, we compared the methylation status of T cells from older and younger mice via an unbiased antibody-based flow cytometric method (Delaney *et al*., 2012). As expected, T cells from older mice had significantly lower methylation relative to T cells from younger mice. CD3^+^ and CD4^+^ T cells from old mice showed ~20% and ~25% lower levels of DNA methylation, respectively, in comparison with young mice (Fig. 1A–B).

### Regulatory T-cell number and FoxP3 expression is higher in older mice relative to younger mice-

We compared the number of CD4^+^ CD25^+^ FoxP3^+^ Treg cells between young and old mice by FACS analysis. Older mice had ~30% higher Tregs as a proportion of total CD4 cells relative to young mice (Fig. 1C). We interrogated if higher Treg number could be explained by differences in FoxP3 expression between young and aged mice. FoxP3 mRNA was ~450% higher and protein was ~45% higher in old CD4^+^ T cells relative to young CD4^+^ T cells (Fig. 1D, E).

### FoxP3 enhancer but not promoter is hypomethylated in older mice

**Figure 2:**
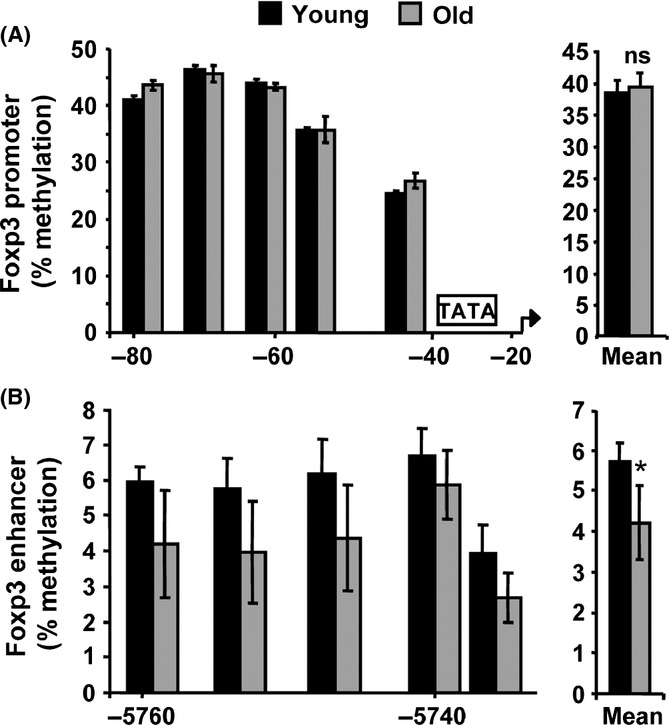
FoxP3 enhancer is hypomethylated during normal aging process. gDNA from CD4^+^ T cells were isolated and of FoxP3 enhancer (A) and promoter (B) regions were subjected to methylation analysis via pyrosequencing as described in Methods. Results are mean ± SEM of 4 independent experiments, each experiment using cells pooled from at least three animals per age group; *P < 0.05.

Several enhancer and promoter regions have been associated with regulation of FoxP3 expression, and de-methylation of the CpG sites within these regions is linked to increase FoxP3 expression coupled with acquisition of a suppressive phenotype (Lal & Bromberg, 2009). To assess whether differences in FoxP3 expression in aged animals could be explained by a differential methylation status of FoxP3 enhancer and/or promoter regions, we performed pyrosequencing across these regions. As shown in Fig. 2A, while the methylation status of the proximal promoter showed no difference, the enhancer methylation status at all the interrogated sites is lower in old CD3^+^ T cells with a 26% mean reduction in comparison with young mice (Fig. 2B).

### Treg from aged mice exhibit greater suppression of Teff proliferation *in vitro*

**Figure 3:**
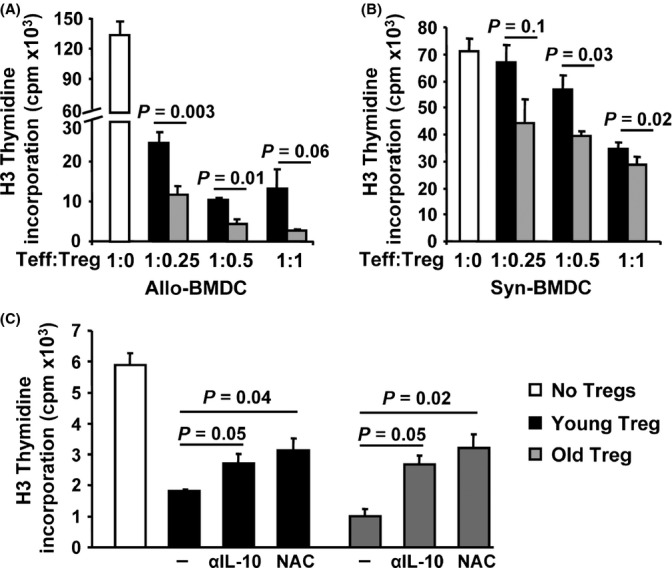
Tregs from aged mice exhibit greater suppression of Teff proliferation *in vitro*. Flow sorted CD4^+^ CD25^+^ Treg cells were incubated with CD4^+^ CD25^−^ Teff cells and either CD11c^+^ allogeneic (A) or syngeneic (B) BMDC at indicated ratios for ~5 days in round bottom 96-well plates. Media were supplemented with 1 μg mL^−1^ anti- CD3e (BD) when coculturing syngeneic BMDC. (C) To confirm the role of IL-10 or redox metabolites in Treg-mediated suppression of Teff proliferation, Treg were incubated with Teff cells at a 1:1 ratio in the presence of DCs. Anti-IL10 antibodies or N-acetyl cysteine (NAC), when used, were administered as a bolus at a final concentration of 10 μg mL^−1^ or 2 mm respectively. Prior to harvesting, cells were incubated overnight in the presence of 3-H thymidine, and incorporation of radioactivity was measured. Results are mean ± SEM and are representative of three (syngeneic) or two (allogeneic) experiments performed in quadruplet, each experiment using cells pooled from at least three animals per age group.

Higher FoxP3 expression would be expected to lead to higher Treg activity. To check the functional consequence of higher Treg levels on Treg-mediated suppression of Teff proliferation, we performed a series of *in vitro* suppression assays. We cultured Tregs from young or old mice with wild-type BL6 Teffs in the presence of allogeneic or syngeneic BMDCs. Treg from old mice exhibited greater suppression of Teff proliferation than Tregs from young mice (Fig. 3A, B). To verify the role of IL-10 or redox mechanism in Treg-mediated suppression of Teff proliferation, we performed the suppression assay in the presence and absence of either anti-IL10-neutrilizing antibodies or NAC. As shown in the Fig. 3C, both anti-IL10 antibodies and NAC reduces the Treg-mediated suppression by young and old Tregs. We also observed an elimination of greater suppressive capacity of old Treg by both the molecules. The effects of NAC or anti-IL10 antibodies were dose-dependent (data not shown).

### Treg from aged mice exhibit greater redox microenvironment perturbation

**Figure 4:**
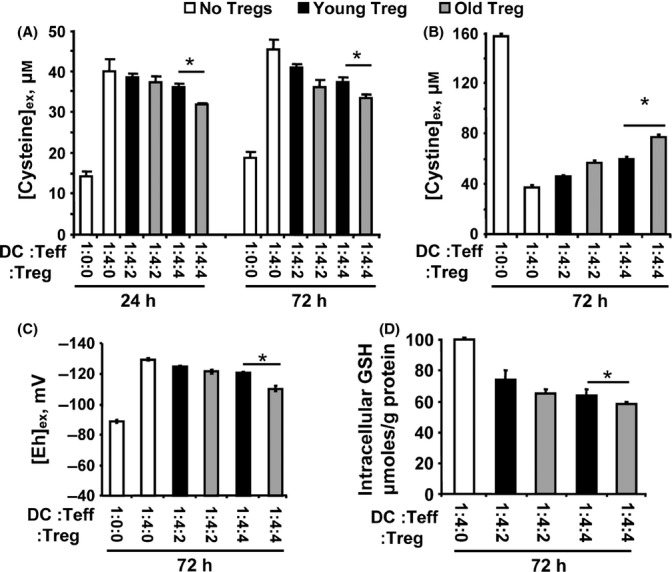
Tregs from aged mice differentially perturb the extracellular redox microenvironment. DCs were cocultured with Teff cells in the absence or presence of Tregs from young or aged mice. Cysteine (A) and cystine (B) concentrations in the conditioned media were quantified via HPLC, and the redox potential (C) was calculated using the Nernst equation (Eh = Eo + RT/2F ln [(cystine)/(cysteine)2], using Eo = −250 mV (pH = 7.4). T cells were separated after 72 h of coculture by gentle washing with PBS and used for intracellular GSH analysis (D) by HPLC. GSH levels were normalized to protein concentrations in each sample. Results represent the mean ± SEM and are representative of four experiments performed in triplicate, each experiment using cells pooled from at least three animals per age group; *P < 0.05.

There are several mechanisms by which Tregs are believed to act to suppress the proliferation of Teff cells. Disruption of redox remodeling by DCs is one mechanism by which Tregs suppress Teff proliferation (Yan *et al*., 2009, 2010). The cysteine/cystine redox potential is a key determinant of the extracellular redox poise (Yan & Banerjee, 2010). Hence, we measured extracellular cystine consumption and cysteine accumulation at two time points (Fig. 4). As expected, conditioned media had higher levels of cysteine when DCs were cocultured with Teffs than cultured alone. The extracellular cysteine concentration decreased in the presence of Tregs (Fig. 4A). When we compared the levels of extracellular cysteine in the presence of Tregs, we found that Tregs from aged mice exhibited greater suppression of cysteine accumulation in a time and DC/Teff/Treg ratio dependent manner (Fig. 4A). The level of extracellular cysteine was ~15% lower in the presence of Tregs from old mice vs. younger mice after 72 h. Extracellular cysteine is derived from cysteine consumed from the culture medium, which is subsequently converted by DCs to GSH, then extruded and hydrolyzed to its component amino acids. The resulting cysteine is taken up by activated Teffs (Yan & Banerjee, 2010). In fact, greater inhibition of cysteine accumulation by Tregs from old mice was accompanied with lower cystine consumption from the media (Fig. 4B). The extracellular redox potential is estimated to be about −80 mV for DC cultured alone, which is associated with growth arrest and differentiation (Yan *et al*., 2009). We measured a redox potential of −85 mV in DC conditioned media after 72 h of culture. Coculture of DCs with Teff cells lowered the redox potential to −130 mV, but in the presence of young Tregs, the redox potential increased to −120 mV. In contrast, Tregs from old mice showed a further increase in the extracellular redox potential to −108 mV (Fig. 4C). The shift in redox potential in the oxidative direction is expected to be less conducive for Teff proliferation (Yan & Banerjee, 2010).

Extracellular cysteine is a limiting substrate for GSH biosynthesis, which is essential for T-cell proliferation (Yan *et al*., 2010). One of the expected consequences of lower extracellular cysteine levels is reduced intracellular GSH level in T cells, and indeed, Teffs exhibited lower intracellular GSH levels in the presence of Tregs. This inhibition was even greater when T cells were cocultured with Treg from old mice relative to cocultures with young Tregs (Fig. 4D).

### Tregs from aged mice accumulate higher IL-10 levels

**Figure 5:**
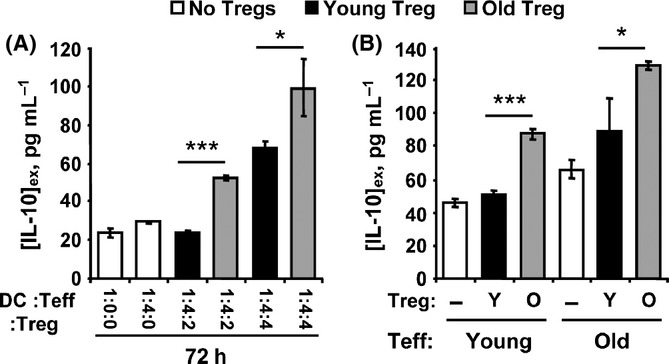
Old Tregs produce higher IL-10 in the conditioned media relative to young Tregs. (A) Treg from young and old mice were cocultured with DC and Teff as 1:4:2 and 1:4:4 (DC/Teff/Treg) ratio in the presence of anti-CD3 antibody for 72 h. (B) Treg from young and old mice were cocultured with Teff from young and old mice at 1:1 ratio in the presence of plate-bound anti-CD3 and soluble anti-CD28 antibody. After 72 h, conditioned media were collected, and IL-10 concentration was measured. Results are mean ± SEM and are representative of two independent experiments performed in triplicates. Y = young Tregs, O = old Tregs. Each experiment used cells pooled from at least three animals per age group; *P < 0.05, ***P < 0.001.

Tregs control Teff cells in both a contact-dependent and contact-independent manner using cytokines such as IL-10, TGF-β, or IL-35 (Vignali *et al*., 2008). To assess whether observed higher suppression by Tregs from old mice is accompanied with higher IL-10 release, we measured the IL-10 concentration in conditioned media. Treg from old mice, when cocultured with DC and Teff cells, produced 100 and 47% more IL-10 in the conditioned media at 1:4:2 and 1:4:4 ratios, respectively, relative to Tregs from young mice (Fig. 5A). To rule out a potentially confounding influence of DCs, we cocultured the Treg from young and old mice with Teffs from young and old mice in the presence of plate-bound anti-CD3 and soluble anti-CD28 and checked the concentration of IL-10 in the conditioned media. Although IL-10 levels were increased when young or old Teff were cocultured with Treg from young mice, the IL-10 levels were significantly higher when the Teffs cells were cocultured with old Tregs (Fig. 5B), suggesting greater suppressive function in old Tregs compared with their young counterparts.

### Tregs from aged mice exerts higher suppression of CD86 on DCs

**Figure 6:**
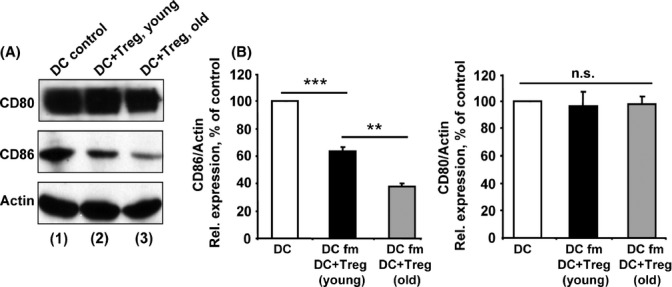
Old Treg exerts higher suppression of CD86 on DCs relative to young Treg. (A) DCs were cocultured with Tregs at 1:4 ratio from young or aged mice for 72 h. Cells were harvested to check the expression of CD86, CD80 on DCs via Western analysis. (B) show the quantification of CD86 and CD80 which is normalized to actin. Results are mean ± SEM and are representative of two independent experiments, each experiment using cells pooled from at least three animals per age group; **P < 0.01, ***P < 0.001, n.s. = not significant.

Tregs downregulate the expression of the costimulatory molecule CD86 on DCs (Cederbom *et al*., 2000). Suppressing CD86 expression interferes with the interaction between CD28 (on Teff cells) and CD86 (on DCs), directing T cells toward a hyporeactive state. While Tregs from neither age group had an effect on the expression of CD80 on DCs, Tregs from aged mice lowered CD86 expression ~40% more than Tregs from young mice (Fig. 6A,B).

## Discussion

Tregs exert their suppressive effects through multiple mechanisms, some that are contact-dependent and others that are contact-independent (Vignali *et al*., 2008).

Using transwell dual chamber assays, Thornton and Shevach showed that physically separated Tregs were unable to suppress Teff proliferation (Thornton & Shevach, 1998). However, suppressive cytokine release of IL-10 (Roncarolo *et al*., 2006), TGF-β (Coffman *et al*., 2009), and IL-35 (Collison *et al*., 2007), as well as IL-2 depletion (Thornton & Shevach, 1998; Pandiyan *et al*., 2007), have been shown to mediate suppression of Teffs without direct contact. Further, Tregs are known to suppress proliferation by interacting with APCs, interfering with costimulatory signals (Cederbom *et al*., 2000; Misra *et al*., 2004; Tang *et al*., 2006) or depleting metabolites (Fallarino *et al*., 2003; Yan *et al*., 2010). We have investigated several mechanisms of Treg-mediated suppression within the context of aging. We report that aged Tregs suppress proliferation more effectively via several distinct mechanisms. Tregs from aged mice, as compared to young mice, release more IL-10, are more effective at downregulating CD86 expression in DCs, and modulate both the extracellular redox environment and intracellular GSH concentration in Teffs, creating conditions that are less favorable to proliferation (Supplementary Fig. S1).

Treg populations increase with aging as a percentage of total T cells (Gregg *et al*., 2005). In BALB/c mice, this increase in Tregs induces tolerance to certain cancers and that cytotoxic anti-tumor activity can be restored *in vivo* via depletion of Tregs (Sharma *et al*., 2006). We confirmed previous studies by showing a significant increase in Tregs in aged mice relative to controls. The so-called ‘inflamm-aging’ hypothesis to explain why Treg numbers increase with aging invokes an interplay between low-grade inflammation over time and an increase in oxidative stress with the development of chronic degenerative conditions like atherosclerosis, diabetes, and obesity (Cannizzo *et al*., 2011). Given the higher levels of inflammatory signals in aging organisms, more Tregs may be necessary to suppress this positive feedback loop of inflammation and disease progression.

Aging is also associated with T-cell’s DNA hypomethylation, an observation that we have confirmed in our study. Lower T-cell DNA methylation is a hallmark of several diseases including systemic lupus erythmatosis and rheumatoid arthritis (Richardson, 2002; Gowers *et al*., 2011; Karouzakis *et al*., 2011). As T cells age and acquire a more autoreactive phenotype, it is reasonable to assume that the body responds by increased production of Tregs to prevent autoimmunity. Key regulatory elements upstream of the FoxP3 gene are sensitive to DNA methylation (Lal & Bromberg, 2009). We demonstrated that FoxP3 expression increases in aged T cells at the RNA and protein level and coincides with an increase in the Tregs population in the proportion to total CD4^+^ T cells. We also demonstrated for the first time that increased FoxP3 expression is accompanied by demethylation of the FoxP3 upstream enhancer. We posit that the aging-associated DNA hypomethylation-driven increase in FoxP3 expression constitutes a strategy for keeping autoimmunity in check as organisms age.

While conferring benefit in ameliorating autoimmunity, a higher Treg population could enhance age-related immune senescence. Classic senescence in CD4 cells features the loss of expression of CD28 costimulatory molecule in addition to shorter telomere length (Effros, 1997) and decreased production and use of IL-2 (Nagel *et al*., 1988). In mice, the pool of CD45RA^+^ naïve T cells decreases due to thymic involution (Nikolich-Zugich, 2008), while the CD45RO^+^ memory compartment and Tregs increase with aging (Han *et al*., 2009). Immunosenescence can lead to poor response to pathogens and tumors (Cannizzo *et al*., 2011). Tregs can promote the immunosenescent phenotype both via interactions with Teff cells and by inhibition of the APC/Teff interaction (Vignali *et al*., 2008). DCs activate Teff cells through engagement of CD3 receptor with the MHCII/antigen complex and CD28 with costimulatory molecules CD80 and CD86. Interestingly, exposure of DCs to hypomethylated DNA collected from aged individuals enhances expression of costimulatory molecules CD80 and CD86 (Agrawal *et al*., 2010). We find that Tregs from aged animals, whose greater numbers may be attributable to hypomethylation of the FoxP3 enhancer, are more effective at interfering with DC/Teff costimulation by downregulation of CD86. Tregs reportedly limit CD86 expression, but not CD80 on DCs via secretion of IL-10 (Slavik *et al*., 1999; Cederbom *et al*., 2000), an observation that we have confirmed. This is consistent with increased IL-10 secretion by Tregs from aged mice effectively suppressing Teff expansion *in vitro*.

Cytokines play a key role in both activation and suppression of T-cell proliferation. Tregs can deplete IL-2 via CD25HI expression and release suppressor cytokines TGF-β and IL-35 in addition to IL-10 (Shevach, 2009). While we did not find a difference in IL-2 concentrations available in DC/Teff/Treg cocultures (data not shown), we found a significant increase in extracellular IL-10 concentration in the presence of Tregs from aged mice. A similar increase in IL-10 was observed during coculture of CD4^+^ CD25^−^ T cells with aged Tregs in the absence of DCs, suggesting that the greater IL-10 release is not dependent on the presence of DCs. Different subsets of Tregs can mediate suppression in different ways, depending on the microenvironment. For example, IL-10 is particularly associated with Tregs that suppress Th17 proliferation (Tian *et al*., 2012). Whether the observed increase in IL-10 secretion by Tregs from old mice indicates a shift in Treg subset populations as part of the aging process, is an intriguing question that remains to be examined.

T-cell activation and proliferation requires a reducing extracellular microenvironment (Angelini *et al*., 2002). Tregs create conditions that are less favorable for Teff proliferation by decreasing DC-derived extracellular cysteine, which inhibits Teff GSH synthesis, thereby interfering with DNA synthesis necessary for clonal expansion (Yan *et al*., 2009, 2010). Tregs from both young and old mice deprive Teffs of extracellular cysteine, raising the redox potential. Our studies reveal that aged Tregs are more effective at inhibiting cystine metabolism by DCs in comparison with young Tregs. The perturbed cysteine/cystine redox potential resulting from higher Treg activity, leads to lower GSH synthesis in Teffs, which in turn lowers their proliferative potential. Greater Treg-mediated redox perturbation might be an important contributing mechanism for immune senescence in the elderly. We have also tested the significance of redox potential by performing suppression assays in the presence of N-acetylcysteine (NAC). NAC supplementation provides additional precursors to glutathione synthesis, enhancing seceretion of cysteine via the γ- glutamyl pathway. This pathway allows provision of cysteine into the extracellular environment, which creates a more favorable redox environment for proliferation. Similar to anti-IL-10 antibody, NAC partially alleviate the Treg-mediated suppression by young and old Tregs. These data suggest that NAC is playing a role in the increased regulatory function of old Tregs. As there are several mechanisms of Treg-mediated suppression of T-cell proliferation, it is not surprising that neither IL-10 neutralization nor NAC by itself can fully prevent suppression.

In summary, our findings suggest that Tregs from aged mice have greater suppressive capability than Tregs from young mice. Aged Tregs exercise greater suppression through multiple known mechanisms of action against both DCs and effector T cells. These mechanisms include downregulation of CD86 on DCs, greater release of IL-10, and lowering of the proliferative potential of Teffs via modulation of the redox microenvironment (Supplementary Fig. 1). Increased total numbers of Tregs in aged animals is associated with demethylation of the FoxP3 enhancer region, suggesting a role for DNA hypomethylation with acquisition of a regulatory phenotype, which may have evolved to protect against hypomethylation-induced T-cell autoreactivity. Taken together, these data suggest that an increased Treg compartment with heightened suppressive function may be an important player in the onset of immune senescence during aging.

## Supporting Information

**Fig. S1.** A model for age-associated higher activity of Tregs.

## References

[b1] Agrawal A, Tay J, Yang GE, Agrawal S, Gupta S (2010). Age-associated epigenetic modifications in human DNA increase its immunogenicity. Aging (Albany NY).

[b2] Angelini G, Gardella S, Ardy M, Ciriolo MR, Filomeni G, Di Trapani G, Clarke F, Sitia R, Rubartelli A (2002). Antigen-presenting dendritic cells provide the reducing extracellular microenvironment required for T lymphocyte activation. Proc. Natl Acad. Sci. USA.

[b3] Cannizzo ES, Clement CC, Sahu R, Follo C, Santambrogio L (2011). Oxidative stress, inflamm-aging and immunosenescence. J Proteomics.

[b4] Castellani P, Angelini G, Delfino L, Matucci A, Rubartelli A (2008). The thiol redox state of lymphoid organs is modified by immunization: role of different immune cell populations. Eur. J. Immunol.

[b5] Cederbom L, Hall H, Ivars F (2000). CD4+ CD25+ regulatory T cells down-regulate co-stimulatory molecules on antigen-presenting cells. Eur. J. Immunol.

[b6] Chiu BC, Stolberg VR, Zhang H, Chensue SW (2007). Increased Foxp3(+) Treg cell activity reduces dendritic cell co-stimulatory molecule expression in aged mice. Mech. Ageing Dev.

[b7] Coffman RL, Lebman DA, Shrader B (2009). Transforming growth factor beta specifically enhances IgA production by lipopolysaccharide-stimulated murine B lymphocytes. J. Exp. Med. 1989. 170: 1039–1044. J Immunol.

[b8] Collison LW, Workman CJ, Kuo TT, Boyd K, Wang Y, Vignali KM, Cross R, Sehy D, Blumberg RS, Vignali DA (2007). The inhibitory cytokine IL-35 contributes to regulatory T-cell function. Nature.

[b9] Delaney C, Hoeltzel M, Garg SK, Warner R, Johnson K, Yung R (2012). Maternal micronutrient supplementation suppresses T cell chemokine receptor expression and function in f1 mice. J. Nutr.

[b10] Effros RB (1997). Loss of CD28 expression on T lymphocytes: a marker of replicative senescence. Dev. Comp. Immunol.

[b11] Fallarino F, Grohmann U, Hwang KW, Orabona C, Vacca C, Bianchi R, Belladonna ML, Fioretti MC, Alegre ML, Puccetti P (2003). Modulation of tryptophan catabolism by regulatory T cells. Nat. Immunol.

[b12] Garg S, Vitvitsky V, Gendelman HE, Banerjee R (2006). Monocyte differentiation, activation, and mycobacterial killing are linked to transsulfuration-dependent redox metabolism. J. Biol. Chem.

[b13] Garg SK, Banerjee R, Kipnis J (2008). Neuroprotective immunity: T cell-derived glutamate endows astrocytes with a neuroprotective phenotype. J Immunol.

[b14] Garg SK, Yan Z, Vitvitsky V, Banerjee R (2011). Differential dependence on cysteine from transsulfuration versus transport during T cell activation. Antioxid. Redox Signal.

[b15] Gowers IR, Walters K, Kiss-Toth E, Read RC, Duff GW, Wilson AG (2011). Age-related loss of CpG methylation in the tumour necrosis factor promoter. Cytokine.

[b16] Gregg R, Smith CM, Clark FJ, Dunnion D, Khan N, Chakraverty R, Nayak L, Moss PA (2005). The number of human peripheral blood CD4+ CD25 high regulatory T cells increases with age. Clin. Exp. Immunol.

[b17] Grimble RF (2006). The effects of sulfur amino acid intake on immune function in humans. J. Nutr.

[b18] Han GM, Zhao B, Jeyaseelan S, Feng JM (2009). Age-associated parallel increase of Foxp3(+)CD4(+) regulatory and CD44(+)CD4(+) memory T cells in SJL/J mice. Cell. Immunol.

[b19] Hori S, Nomura T, Sakaguchi S (2003). Control of regulatory T cell development by the transcription factor Foxp3. Science.

[b20] Huehn J, Polansky JK, Hamann A (2009). Epigenetic control of FOXP3 expression: the key to a stable regulatory T-cell lineage?. Nat. Rev. Immunol.

[b21] Karouzakis E, Gay RE, Gay S, Neidhart M (2011). Epigenetic deregulation in rheumatoid arthritis. Adv. Exp. Med. Biol.

[b22] Lal G, Bromberg JS (2009). Epigenetic mechanisms of regulation of Foxp3 expression. Blood.

[b23] Mendez S, Reckling SK, Piccirillo CA, Sacks D, Belkaid Y (2004). Role for CD4(+) CD25(+) regulatory T cells in reactivation of persistent leishmaniasis and control of concomitant immunity. J. Exp. Med.

[b24] Messina JP, Lawrence DA (1989). Cell cycle progression of glutathione-depleted human peripheral blood mononuclear cells is inhibited at S phase. J Immunol.

[b25] Misra N, Bayry J, Lacroix-Desmazes S, Kazatchkine MD, Kaveri SV (2004). Cutting edge: human CD4+ CD25+ T cells restrain the maturation and antigen-presenting function of dendritic cells. J Immunol.

[b26] Nagel JE, Chopra RK, Chrest FJ, McCoy MT, Schneider EL, Holbrook NJ, Adler WH (1988). Decreased proliferation, interleukin 2 synthesis, and interleukin 2 receptor expression are accompanied by decreased mRNA expression in phytohemagglutinin-stimulated cells from elderly donors. J Clin Invest.

[b27] Nikolich-Zugich J (2008). Ageing and life-long maintenance of T-cell subsets in the face of latent persistent infections. Nat. Rev. Immunol.

[b28] Pandiyan P, Zheng L, Ishihara S, Reed J, Lenardo MJ (2007). CD4+ CD25+ Foxp3+ regulatory T cells induce cytokine deprivation-mediated apoptosis of effector CD4+ T cells. Nat. Immunol.

[b29] Raynor J, Lages CS, Shehata H, Hildeman DA, Chougnet CA (2012). Homeostasis and function of regulatory T cells in aging. Curr. Opin. Immunol.

[b30] Richardson BC (2002). Role of DNA methylation in the regulation of cell function: autoimmunity, aging and cancer. J. Nutr.

[b31] Roncarolo MG, Gregori S, Battaglia M, Bacchetta R, Fleischhauer K, Levings MK (2006). Interleukin-10-secreting type 1 regulatory T cells in rodents and humans. Immunol. Rev.

[b32] Sharma S, Dominguez AL, Lustgarten J (2006). High accumulation of T regulatory cells prevents the activation of immune responses in aged animals. J Immunol.

[b33] Shevach EM (2009). Mechanisms of foxp3+ T regulatory cell-mediated suppression. Immunity.

[b34] Slavik JM, Hutchcroft JE, Bierer BE (1999). CD28/CTLA-4 and CD80/CD86 families: signaling and function. Immunol. Res.

[b35] Staal FJ (1998). Glutathione and HIV infection: reduced reduced, or increased oxidized?. Eur. J. Clin. Invest.

[b36] Suthanthiran M, Anderson ME, Sharma VK, Meister A (1990). Glutathione regulates activation-dependent DNA synthesis in highly purified normal human T lymphocytes stimulated via the CD2 and CD3 antigens. Proc. Natl Acad. Sci. USA.

[b37] Tang Q, Adams JY, Tooley AJ, Bi M, Fife BT, Serra P, Santamaria P, Locksley RM, Krummel MF, Bluestone JA (2006). Visualizing regulatory T cell control of autoimmune responses in nonobese diabetic mice. Nat. Immunol.

[b38] Thornton AM, Shevach EM (1998). CD4+ CD25+ immunoregulatory T cells suppress polyclonal T cell activation *in vitro* by inhibiting interleukin 2 production. J. Exp. Med.

[b39] Tian L, Humblet-Baron S, Liston A (2012). Immune tolerance: are regulatory T cell subsets needed to explain suppression of autoimmunity?. BioEssays.

[b40] Vignali DA, Collison LW, Workman CJ (2008). How regulatory T cells work. Nat. Rev. Immunol.

[b41] Yan Z, Banerjee R (2010). Redox remodeling as an immunoregulatory strategy. Biochemistry.

[b42] Yan Z, Garg SK, Kipnis J, Banerjee R (2009). Extracellular redox modulation by regulatory T cells. Nat. Chem. Biol.

[b43] Yan Z, Garg SK, Banerjee R (2010). Regulatory T cells interfere with glutathione metabolism in dendritic cells and T cells. J. Biol. Chem.

