# Supplementary figures and images for: Aging is associated with increased regulatory T-cell function
Source: Aging Cell. 2014 Feb 25;13(3):441–8. doi: 10.1111/acel.12191 (PMC4032602; doi:10.1111/acel.12191)

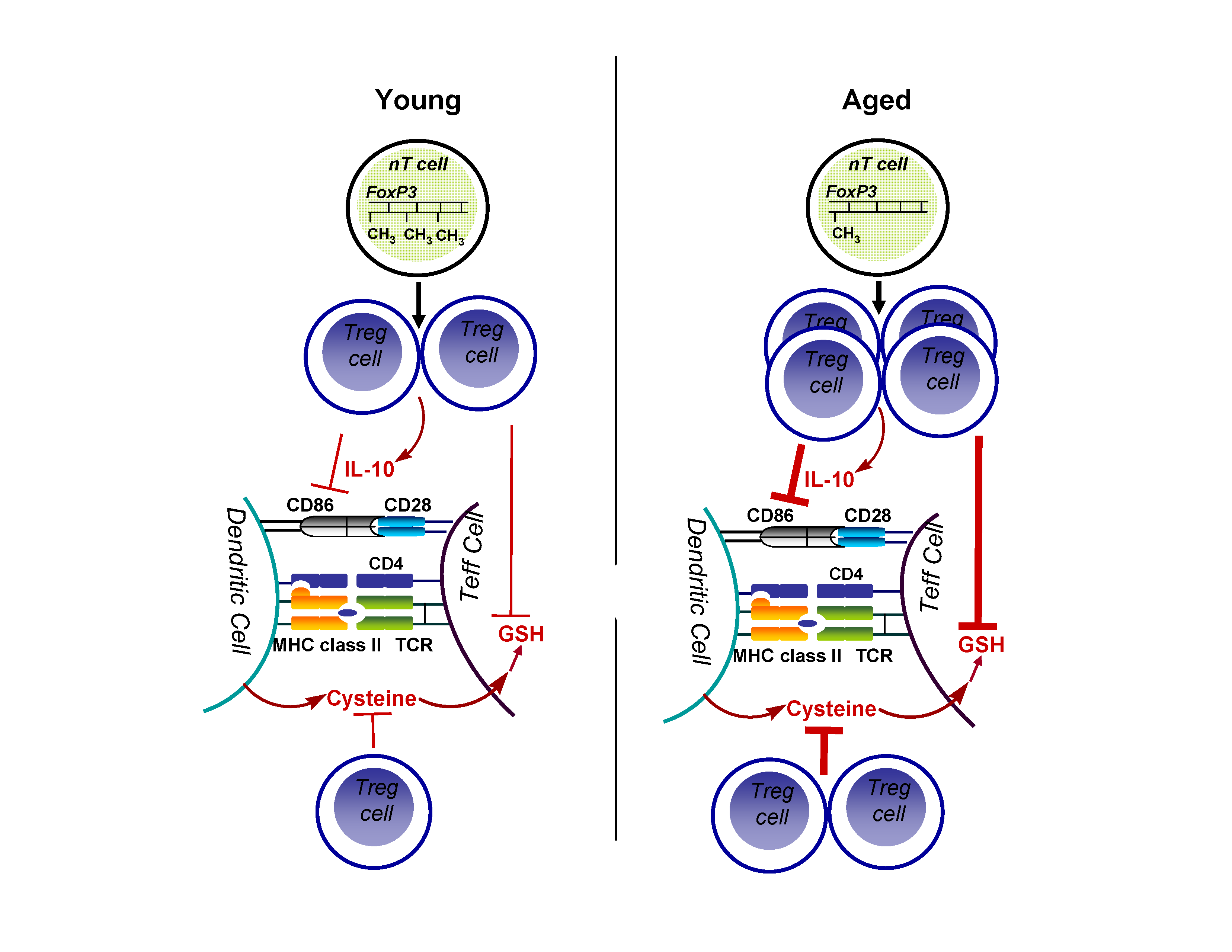

Supplement: Supplementary file 1 — Fig. S1. A model for age-associated higher activity of Tregs. [file acel0013-0441-sd1.tiff]
